# Unobtrusive detection of Parkinson’s disease from multi-modal and in-the-wild sensor data using deep learning techniques

**DOI:** 10.1038/s41598-020-78418-8

**Published:** 2020-12-07

**Authors:** Alexandros Papadopoulos, Dimitrios Iakovakis, Lisa Klingelhoefer, Sevasti Bostantjopoulou, K. Ray Chaudhuri, Konstantinos Kyritsis, Stelios Hadjidimitriou, Vasileios Charisis, Leontios J. Hadjileontiadis, Anastasios Delopoulos

**Affiliations:** 1 Multimedia Understanding Group, Information Processing Laboratory, Department of Electrical and Computer Engineering, Aristotle University of Thessaloniki, Thessaloníki , Greece; 2 Signal Processing and Biomedical Technology Unit, Telecommunications Laboratory, Department of Electrical and Computer Engineering, Aristotle University of Thessaloniki, Thessaloníki, Greece; 3 Department of Neurology, Technical University of Dresden, Dresden, Germany; 4 Third Neurological Clinic, Papanikolaou Hospital, Thessaloníki, Greece; 5 International Parkinson Excellence Research Centre, King’s College Hospital NHS Foundation Trust, London, United Kingdom; 6 Department of Electrical Engineering and Computer Science/Department of Biomedical Engineering, Khalifa University of Science and Technology, Abu Dhabi, UAE

**Keywords:** Parkinson's disease, Statistics, Information technology

## Abstract

Parkinson’s Disease (PD) is the second most common neurodegenerative disorder, affecting more than 1% of the population above 60 years old with both motor and non-motor symptoms of escalating severity as it progresses. Since it cannot be cured, treatment options focus on the improvement of PD symptoms. In fact, evidence suggests that early PD intervention has the potential to slow down symptom progression and improve the general quality of life in the long term. However, the initial motor symptoms are usually very subtle and, as a result, patients seek medical assistance only when their condition has substantially deteriorated; thus, missing the opportunity for an improved clinical outcome. This situation highlights the need for accessible tools that can screen for early motor PD symptoms and alert individuals to act accordingly. Here we show that PD and its motor symptoms can unobtrusively be detected from the combination of accelerometer and touchscreen typing data that are passively captured during natural user-smartphone interaction. To this end, we introduce a deep learning framework that analyses such data to simultaneously predict tremor, fine-motor impairment and PD. In a validation dataset from 22 clinically-assessed subjects (8 Healthy Controls (HC)/14 PD patients with a total data contribution of 18.305 accelerometer and 2.922 typing sessions), the proposed approach achieved 0.86/0.93 sensitivity/specificity for the binary classification task of HC versus PD. Additional validation on data from 157 subjects (131 HC/26 PD with a total contribution of 76.528 accelerometer and 18.069 typing sessions) with self-reported health status (HC or PD), resulted in area under curve of 0.87, with sensitivity/specificity of 0.92/0.69 and 0.60/0.92 at the operating points of highest sensitivity or specificity, respectively. Our findings suggest that the proposed method can be used as a stepping stone towards the development of an accessible PD screening tool that will passively monitor the subject-smartphone interaction for signs of PD and which could be used to reduce the critical gap between disease onset and start of treatment.

## Introduction

Parkinson’s Disease (PD) is a progressive long-term neurodegenerative disease that affects about 7 million people globally^1,2^. In its early stages, it mainly manifests through motor symptoms, such as tremor, bradykinesia and rigidity^3^. Even though the disease itself is incurable, early diagnosis has been linked to improved clinical outcomes, as the early stage symptoms can be managed more effectively, through appropriately targeted medical^4^ or physical^5^ interventions, thus limiting their progression rate and increasing the patient’s chance for an improved quality of life in the long run. Unfortunately, early motor signs of PD are so mild that patients often ignore them, causing the condition to remain under the radar for a critical period of time that could otherwise be exploited. This emphasizes the need for an accessible tool that will monitor subjects remotely, unobtrusively and throughout their daily routine and urge them to visit a doctor if it detects motor symptoms that could be attributed to an early PD onset.

**Figure 1:**
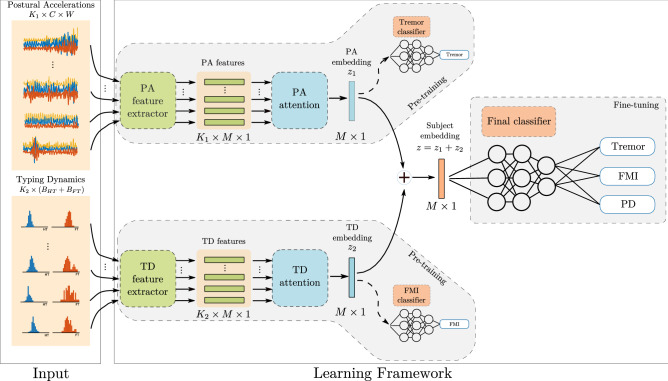
High-level overview of the deep learning system. Both input streams, represented by the Postural Accelerations (PA) and Typing Dynamics (TD) bags, consist of multiple data recordings ($K_1$ and $K_2$, respectively) that are transformed independently using a feature extraction module. The resulting *M*-dimensional features are used to produce a fixed-length embedding (also *M*-dimensional) using an attention-pooling module. The embeddings for each modality are fused together via a sum operation to produce the subject embedding, which is then used as input to a multi-label classifier module that outputs the probability of tremor, fine-motor impairment (FMI) and PD. Initially, the feature extractors and attention modules of the two modalities are separately pre-trained against the respective symptom ground-truth (tremor or FMI), using a temporary single-output classifier module. After pre-training, the initial classifier modules are discarded, the feature extraction and attention modules are frozen, the embedding vectors are joined and a new multi-output final classifier module is introduced and is fine-tuned using a multi-label logistic loss function (Eq. 4). *C* denotes the number of accelerometer channels, *W* the length in samples of each segment in the PA bag and $B_{HT}, B_{FT}$ the number of bins for the hold and flight time histograms. Values for all parameters are given in Methods. Figure was drawn using Inkscape v1.0 https://inkscape.org/.

In current clinical practice, screening a potential patient for PD involves the use of the Unified Parkinson’s Disease Rating Scale (UPDRS)^6^. The latter captures specific aspects of motor behaviour through a structured neurological examination, as well as certain aspects of daily life through a self-evaluation interview. The motor examination (UPDRS Part III) is conducted by a neurologist and contains a series of tests that attempt to quantify, in an increasing scale of 0 to 4, the severity of each item in the PD motor symptomatology. The sum of the individual symptom scores is then used as a total PD score. Subsequent evaluations of the UPDRS at large time intervals, serve as a way of monitoring the longitudinal course of the disease.

Harnessing the sensors embedded in off-the-shelf smart devices for healthcare applications is an emerging area of research, fueled by the proliferation and wide availability of such devices^7,8^. Most works in the literature attempt to infer PD from single symptom cues that are estimated from sensor data collected during tests that require the user’s active participation^9–11^. However, inferring PD from the presence of a single symptom is not reliable, as the disease may manifest with different symptoms in each patient^3^, while the need of active engagement may also negatively affect adherence to the data collection protocol^12^.

In this study, we take a first step towards a data-driven approach for the early detection of PD by developing a screening methodology that is based on how a subject interacts with his/her smartphone during everyday life. Here we aim to detect PD using a multi-symptom approach that merges passively-captured data from two different smartphone sensors via a novel deep learning framework. Our method is inspired by the typical workflow of a neurologist, in the sense that it outputs a score for tremor and fine-motor impairment (FMI), two of the most common PD motor symptoms, as well as a score for PD. To that end, we use two sources of data: the tri-axial acceleration values obtained from the Inertial Measurement Unit (IMU) sensor of the smartphone and the keystroke timing data (press and release timestamps of each keystroke) captured during typing with the smartphone’s virtual keyboard. The accelerometer data are used to monitor the subject for hand tremor, while the typing data are used to detect signs of FMI during typing. Both data sources are captured passively and unobtrusively when the users perform common actions with their phone, i.e., when they place phone calls or when they type across smartphone applications that require typing interaction. For analysing the collected data, we design an end-to-end system based on deep learning^13^, that digests the multi-modal set of signals contributed by a subject and reduces it to a single fixed-length vector that is then used for multi-label subject classification. More specifically, the learning system digests the subject-smartphone interaction data and predicts the probability that the subject has tremor, FMI and PD. In this framework, each subject is represented by his/her Postural Accelerations (PA) and Typing Dynamics (TD) bags (unordered sets of data instances). The PA bag consists of tri-axial acceleration signal segments of fixed length, while the TD contains the keystroke timing dynamics of each typing session, in the form of the Flight Time (FT) (the time between releasing a key and pressing the next) and the Hold Time (HT) (the time interval between pressing and releasing a key) histograms for all the keystrokes in a session. The network processes these inputs via a sequence of feature extraction, attention pooling and classifier modules to ultimately transform them into probabilities for tremor, FMI and PD. A high-level overview of the overall system is presented in Fig.  1.

Our approach differs from all the works in the related literature (described in detail in Section Discussion) in being the first to consider the problem of automated PD detection based on multiple data sources, and covering different PD motor symptoms, that are unobtrusively captured from the user’s personal smartphone during the stochastic conditions of daily living (data in-the-wild). Validation of the proposed approach on two data sets, i.e., one with in-the-wild data from 22 clinically-assessed subjects (8 Healthy Controls (HC)/14 PD patients) and another with in-the-wild data from 157 subjects with self-reported PD status (131 HC/26 PD), justified the efficiency of our approach, reaching 0.93/0.86 sensitivity/specificity and an Area Under Curve (AUC) around 0.87, respectively on each dataset. These encouraging results promote the fusion of ecologically captured data from multiple sources during smartphone use, as an enhanced informative medium for PD screening.

## Results

To evaluate our approach, we perform experiments on two datasets: a small, clinically-validated set of 22 subjects (14 PD patients and 8 healthy controls), for whom clinical scores are provided by movement disorders experts, and a large dataset of 157 subjects, for whom only a self-report of whether they have been diagnosed with PD is available. We will refer to the small dataset as *SData* set and to the large dataset as *GData* set. These terms originate from the two data collection studies that were introduced in the i-PROGNOSIS project^14^ and which generated the datasets used in this work. The demographics of both datasets are presented in Table 1. A detailed account of the data collection methodology is given in Section Collection of the SData and GData sets.

**Table 1:** Demographics and symptom information for the PD patient (PD) and Healthy Control (HC) populations of the two datasets (SData and GData) used in our experiments. Reported symptom scores include UPDRS items 16 (tremor self-report), 20 (hand rest tremor), 21 (hand action tremor), 22 (hand rigidity), 23 (finger tapping) and 31 (body bradykinesia), as well as the sum of all UPDRS part-III items. Scores for UPDRS 20–23 refer to the sum of both hand scores. UPDRS scores are not available for the GData set, as the GData subjects did not undergo a medical examination. The age corrected GData column contains the demographics of a typical random GData subset, sampled in such a way that the mean age of the HC population lies within ± 2.5 years of the mean age of the PD population (note: the maximum value of 12 in the total UPDRS-III score in the Healthy Control population is caused by a subject that exhibits arthrosis and other problems unrelated to PD.) LEDD stands for Levodopa Equivalent Daily Dose.

Count	SData set		GData set		GData set (age corrected)	
	HC	PD	HC	PD	HC	PD
	8	14	131	26	75	26
Age	50.5 (9.0)	60.7 (9.8)	54.5 (10.0)	62.5 (8.9)	60.0 (9.5)	62.5 (8.9)
Gender (female/male)	4/4	3/11	55/76	12/14	35/40	12/14
Years diagnosed (mean, std)	–	7.5 (3.6)				
Years diagnosed [min, max]	–	[1, 20]	Not available			
PD medication intake (yes/no)	0/8	13/1				
LEDD in mg (mean, std)	0 (0)	510.7 (379.9)				
LEDD in mg [min , max]	[0, 0]	[0, 1291]				
UPDRS 16 mean (std)	0.1 (0.3)	1.3 (1.1)				
UPDRS 16 [min, max]	[0, 1]	[0, 4]				
UPDRS 20 mean (std)	0.0 (0.0)	1.4 (1.4)				
UPDRS 20 [min,max]	[0, 0]	[0, 5]				
UPDRS 21 mean (std)	0.0 (0.0)	1.2 (1.9)				
UPDRS 21 [min, max]	[0, 0]	[0, 7]				
UPDRS 22 mean (std)	0.2 (0.4)	2.2 (1.8)	Not available			
UPDRS 22 [min, max]	[0, 1]	[0, 7]				
UPDRS 23 mean (std)	0.2 (0.6)	2.2 (1.5)				
UPDRS 23 [min, max]	[0, 2]	[5, 5]				
UPDRS 31 mean (std)	0.2 (0.4)	1.2 (0.4)				
UPDRS 31 [min, max]	[0, 1]	[1, 2]				
Total UPDRS-III (mean, std)	1.7 (2.5)	21.1 (15.0)				
Total UPDRS-III [min, max]	[0, 12]	[3, 62]				

In the first experiment, we employ a Leave One Subject Out (LOSO) validation scheme on the SData set. In a given LOSO iteration, we set aside the data of an SData subject and train a model using the data of the rest. The trained model is then used to predict the label of the left-out subject. The process is repeated so that all SData subjects are used for validation exactly once. The classification metrics of each LOSO iteration are collected and the average classification performance across them is measured and reported. The resulting metric is a nearly unbiased estimator of the model’s probability of error^15^. To account for the inherent randomness in neural network initialisation, we repeat model training 10 times for each left-out subject. We report the average performance metrics from this experimental setup in Table 2. We also compare the average performance of the fused model in the tasks of tremor and FMI detection, with the standalone symptom models that emerge during the single-label pre-training step of the unfused model branches. As we can see by comparing the two distinct parts of Table 2 with respect to the Tremor and FMI targets, the joint training of the fused model with the multi-label target seems to improve the average performance of the individual symptom detection ( average improvement of $\sim \,3\%$ in some performance metrics without deterioration in the rest over all LOSO iterations and random model initialisations), in addition to accurately predicting PD itself. A statistical significance test, comparing the f1 scores between the 2 models, showed that the fused model is significantly better at tremor prediction with $p < 0.001$ and better at FMI prediction with $p=0.12$. However, owing to the small number of samples for the *t*-test, these *p* values should be interpreted with caution.

**Table 2:** Classification performance for the LOSO experiment on the SData set. For each predicted label (tremor, FMI and PD) we report the average sensitivity, specificity and precision across 10 independent experimental trials. The first two rows present the predictive performance of standalone symptom classifiers. Such models arise during the initial pre-training step, when the separate branches that comprise the total model are separately trained against a single symptom label (the pre-training procedure is described in detail in Section Training details). The rest of the table, presents the performance of the fused multi-label classifier.

Classifier	Target	Sensitivity	Specificity	Precision
Tremor	Tremor	0.827	0.909	0.901
FMI	FMI	0.866	0.800	0.902
Fused multi-label	Tremor	0.854	0.936	0.930
	FMI	0.866	0.842	0.922
	PD	0.928	0.862	0.921

**Figure 2:**
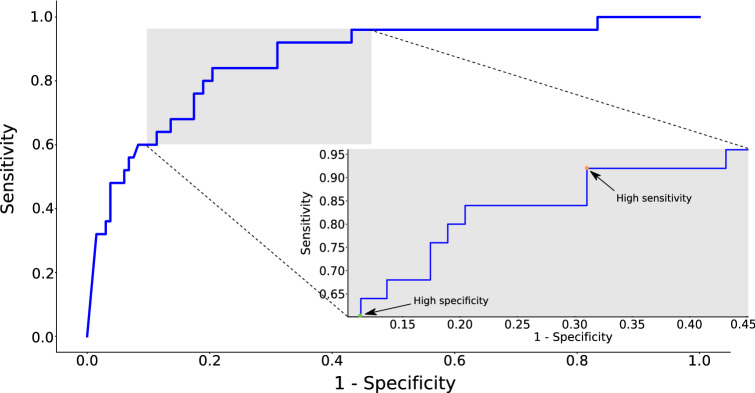
ROC curve obtained by using an ensemble of 10 models trained on the SData set ($n=22$), to infer PD on the GData set ($n=157$). The corresponding AUC is 0.868 with a $95\%$ confidence interval of 0.773–0.948. Figure was drawn using Inkscape v1.0 https://inkscape.org/.

For the second experiment, we train an ensemble of 10 models using all the subjects from the SData set and use it to predict the PD status of the subjects in the GData set. We use the average score of the individual models of the ensemble as the PD score for a subject. We summarise the ensemble’s performance by means of the *Area Under Curve (AUC)* metric. This resulting *Receiver Operating Characteristic (ROC)* curve is depicted in Fig.  2. The AUC score along with the sensitivity/specificity metrics at the operating points of high sensitivity and high specificity are presented in Table 3. The high sensitivity operating point is usually selected if high positive prediction accuracy is more desirable than the need of minimising false positives. For example, a system that aims to identify as many PD patients as possible without worrying about scaring otherwise healthy users, will operate at the high sensitivity point. On the other hand, the high specificity point is preferred if limiting the number of false positives is more important. For example, a system that intends to alert subjects only when it is very confident of its prediction, will operate at the high specificity point.

As we can see in Table 1, the mean age of the healthy controls is significantly lower than the PD population. Age is an important covariate for PD, whose influence must be eliminated in the experimental procedure, in order to ensure that the resulting model indeed detects PD and not some proxy of age. To mitigate this issue, we performed an additional experiment, in which we randomly generated age-matched (within a tolerance of 2.5 years) subsets of the GData set and computed the mean AUC across them. The average AUC for 10 of such subsets is given in the relevant column of Table 3. We limit this procedure to the GData set, as it contains a larger number of subjects and, therefore, allows us more flexibility in generating appropriate subsets for testing. However, we believe that the fact that a model which was trained on the age-imbalanced SData set, generalises well on the age-corrected GData set, serves as adequate proof. Finally, this conclusion is also supported by the fact that using age as a PD predictor in the GData set, yields an AUC of $\sim 0.71$ (with a $95\%$ CI of 0.588–0.814), that is significantly less than the AUC of $\sim 0.87$ achieved by the model.

**Table 3:** Experimental results of an ensemble of 10 fused multi-label models trained on the SData set ($n=22$) and evaluated on the GData set ($n=157$) for the detection of PD. The AUC in the age corrected GData column refers to the mean AUC across 10 randomly generated, age-matched GData subsets (as such, we do not report performance metrics values at the two operating points for this experiment).

Operating point	Metric	GData	GData (age corrected)
High sensitivity $(>0.9)$	Sensitivity	0.920	
	Specificity	0.689	
High specificity $(>0.9)$	Sensitivity	0.600	
	Specificity	0.917	
	AUC	0.868	0.834

**Figure 3:**
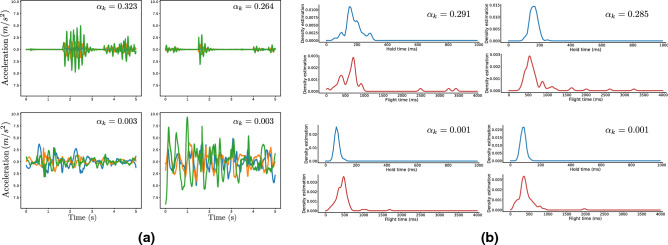
Visualization of bag instances that receive the highest and lowest attention weights by the proposed model. Figures were drawn using Inkscape v1.0 https://inkscape.org/. (**a)** View of the tri-axial (x-axis: blue, y-axis:orange, z-axis: green) accelerometer segments corresponding to the two highest (top row) and two lowest (bottom row) attention weights assigned by the model, for a PD subject that exhibits tremor. As we can see, the model assigns larger attention weights ak to the acceleration segments that contain sinusoidal components in the PD tremor frequency band of 3–8Hz (top row) and low weights to segments that lack such patterns (bottom left) or whose frequency content is outside the target frequency band (bottom right). (**b**) View of the hold and flight time histograms (smoothed for visualization purposes via a kernel-density estimator) corresponding to the two highest (top row) and two lowest (bottom row) attention weights for a PD subject that exhibits FMI. Notice how the model assigns large attention weights to typing sessions in which the hold and flight time distributions are multi-modal and shifted towards regions of slower typing, in contrast to the sessions that receive little attention and in which the distributions are unimodal and located at regions of faster typing.

Finally, our method provides an elegant way of visualising the instances of a bag that contain the relevant symptom. The attention mechanism that is used for pooling the individual data instances of a bag into a single vector representation, (a process described in detail in “Problem formulation” section ) assigns a different weight to each data instance, so that instances with large weights contribute more to the resulting representation. By examining the instances that receive large weights versus those that receive smaller weights, we can identify which of the instances in the bag contain the relevant symptom and which are symptom-free. We provide examples of such visualizations for both the PA and the TD bag of a subject in Fig. 3a,b respectively.

## Discussion

The problem of automated PD detection has received much attention in recent years. A wide range of sensors have been suggested to capture specific aspects of the PD-related symptomatology, such as IMU sensors for detecting gait alterations^16–19^ and hand tremor^10,20–23^, microphones for identifying incidents of speech impairment^24–27^, keyboards (mechanical or virtual)^11,28,29^ for detecting rigidity and bradykinesia, and writing equipment^9,16,30^ for estimating the level of fine motor impairment.

The common denominator in all these studies is that they attempt to infer PD from single symptom cues. This is inherently problematic, since PD manifests differently in different subjects^3^. Consequently, subjects that do not exhibit the targeted symptom, but are still PD positive, are bound to be missed by such a system. This naturally leads to the conclusion that, in order to have a high-performing PD detection system, the fusion of multiple sources of information, covering different PD symptoms, is necessary. Unfortunately, in this case the research landscape is rather desolate, with only a handful of works addressing the problem in such perspective. For instance, one very early work^16^ suggested to combine handwriting and gait features, leading to improved PD classification performance over the unfused features. A more recent work^31^ introduced a method based on multi-view representation learning to jointly learn handwriting and gait features and use them alongside speech-based features to improve PD classification and prediction of the patient neurological state. This idea was further developed in^32^ and an approach to combine speech, handwriting and gait information was proposed based on training separate Convolutional Neural Networks (CNNs) for each modality and then merging features from the last hidden layer of each CNN, to produce a single feature vector for each subject. The resulting vector was ultimately used as input to a non-linear Support Vector Machine (SVM) that performed the final classification.

Our work here also follows a multi-modal approach towards PD detection, yet significantly differs from previous works in a number of ways. In particular: i) In many previous works, data collection took place through the use of specialised equipment, such as body-worn sensors for capturing gait information^17,32^, physical keyboards for capturing keystroke dynamics^11^ and drawing tablets^32^ or even pen and paper^30^ for collecting handwriting data. Our approach, on the other hand, captures PD-related data by utilising the IMU sensor and the virtual keyboard embedded in the subjects’ personal smartphones. ii) The subjects in almost all previous works had to follow scripted self-initiated data capture scenarios, such as performing specific actions, like writing or resting for some minutes^20,21,33^, pronouncing specific words and sentences^25,34^, drawing specific shapes^9,30^ or transcribing given text excerpts^11,28^. Apart from our two previous works^23,29^, that collect uni-modal data in-the-wild, only one other work^35^ considers the unscripted data collection problem. However, it does so limited to a home environment and involves very low scale experiments (as little as 2 PD patients), while also employing a custom-made body-worn system of accelerometers and motion sensors. Our approach differs from all the existing literature, by being the first to consider the problem of simultaneously detecting PD and its symptoms from multi-modal data captured unobtrusively and in-the-wild, using the readily available sensors of modern smartphones. The combination of these properties facilitates user engagement and enables the development of a remote PD screening tool that can be deployed in large-scale and for long time periods.

To that end, we proposed an end-to-end trainable deep learning framework, consisting of independent building blocks, such as feature extractors and attention pooling modules, that outputs binary predictions for tremor, FMI and PD. The introduction of attention offers an elegant way of modelling the fluctuating nature of some PD symptoms, which can be caused by symptom-alleviating medication or can be inherent to some symptoms (for instance tremor increases in stressful situations and alleviates in relaxed situations). In addition, by visualising the instances in a data bag that have received large attention weights, we can identify the specific data that contributed to the model’s predictive functionality, thus, adding a degree of interpretability to the approach. We provide examples of such visualizations for both the acceleration (Fig. 3a) and the typing modalities (Fig.  3b).

The resulting model achieved $92.8\%$ sensitivity and $86.2\%$ specificity for PD detection in a LOSO experiment with 22 subjects, averaged over 10 random trials, while also performing equally well for the tasks of tremor and FMI detection (Table 2). We also showed some evidence that the joint multi-label training objective is beneficial for the secondary symptom detection tasks as well, based on the difference in performance between the initially pre-trained symptom classifiers and the final multi-label model. To account for the small number of subjects in the first experiment, we extended our analysis to a larger dataset of 157 subjects, where we showed that an ensemble of 10 models trained on the initial 22 subjects, was able to generalise well, achieving an AUC of 0.868 (with a $95\%$ confidence interval (CI) of 0.773–0.948) over the total dataset and an average AUC of 0.834 over 10 random age-matched subsets (Table 3), for the task of PD detection. In general, while the results of both experiments are very encouraging, future work will aim for a larger set of newly-diagnosed PD patients, as well as an enriched pool of healthy controls, so that the model’s classification performance can be estimated more accurately for subjects at the early PD stages or for healthy controls with PD-like symptoms, such as essential tremor.

Aside from its performance, one of the most attractive properties of the proposed framework is its extendability, i.e., due to its modular structure, it is rather straightforward to incorporate additional data modalities under the same architecture. For instance, a third branch that would process speech information, using separate feature extraction and attention modules, could be readily added without compromising the model’s end-to-end nature, while reaping the apparent benefits of the joint multi-modal training scheme. In addition, fusing the different modalities together by summing their embeddings, makes sure that the dimensionality of the input to the final classifier does not increase as new modalities are added (which would be the case if we concatenated the embeddings). Apart from keeping the dimensionality low, the most important aspect of this design choice is that it theoretically enables us to perform training and inference in the presence of missing data; hence, should a subject lacks data for one or more modalities, we can simply compute his/her final embedding by summing the embeddings of the modalities for which s/he did provide data. While definitely not optimal, this procedure allows us to utilise any incomplete data that would otherwise be unusable.

A limitation of our approach is that, in its current form, it may lead to false positive PD predictions even in cases where the symptom detection is performed correctly. In fact, it is natural for a healthy subject to exhibit some symptom from the PD motor symptomatology. For example, tremor is associated with many other conditions and in some cases it may not even be caused by pathological factors^36^ (e.g., physiological tremor). However, should that single symptom be detected, our model will classify such subjects as having PD. This emanates from the relatively small size of the training set, in which almost all the healthy controls, in addition to being PD negative, did not exhibit any of the examined PD symptoms. As such, the model is likely to associate even a single symptom with a positive PD case. As far as we can see, there are two main directions of improvement, that could alleviate the impact of this issue: a) acquisition of new training examples that cover these specific cases, and b) incorporation of additional data modalities, so that any predictions are based on a more complete view of the subject. As these directions are complementary to each other, they can be pursued independently.

A more technical problem arises as more data are collected per subject. At the moment, the data contribution of a subject is transformed into bags of a fixed maximum size. The bag size parameter is large enough so that the produced bags contain most of the contributed data. In addition, filtering mechanisms are used to reduce the effective bag size (Sections Postural Accelerations bag creation, Typing Dynamics bag creation). These mechanisms implement some ranking criterion and are used to discard instances that are deemed to be of little interest. However, as the collected data increase, the amount of instances that is discarded, in order to keep the bag size fixed, also becomes greater. This can eventually lead to loss of significant information. For example, for a subject with mild symptoms and a large data contribution the resulting bag may ultimately not contain the informative sessions, due to the max bag size constraint. On the other hand, increasing the bag size imposes a substantial computational overhead during both training and inference. The development of more robust filtering mechanisms during the bag creation step, could help mitigating this issue. Alternatively, we could consider a reformulation of the problem, in which each subject participates with multiple data bags. In such a case, the attention mechanisms would be applied in two levels: both within each bag (as is the case currently) and across the different bags (as some bags may be completely symptom-free). Although this approach would severely complicate the model architecture, we believe it could serve as a viable alternative.

We have presented a framework for the remote detection of PD, tremor and fine-motor impairment based on smartphone-derived sensor data captured completely unobtrusively and in-the-wild. We believe our work is the first to address the problem of detecting PD and its symptoms from multi-modal data under these conditions. Performance-wise, our approach produced good classification results in two real-world datasets of varying size. The discussion of the previous paragraphs outlined an abundance of potential future work, such as the incorporation of new data modalities, like speech, and the adaptation of the framework to handle missing data, as well as specific interventions targeting the system’s week spots. Nonetheless, we believe that the proposed approach is a solid first step towards a high-performing remote PD detection system that will be used to unobtrusively monitor a subject and urge them to visit a doctor if it detects signs of PD.

## Methods

### Postural accelerations bag creation

Prior to the actual training procedure, the sensor data contributed by the subject must be expressed in a form that can be analysed by the network. In the case of the acceleration modality, the data contribution is summarised as a set of fixed-length acceleration signal segments, namely the postural accelerations bag. Transforming the captured raw, variable-length accelerometer recordings to the PA bag involves a pre-processing and a selection step, similar to our earlier work^23^. First, problematic recordings are discarded. As problematic we characterise recordings with: (a) duration less than 20 s, (b) very low sampling frequency $$(<50\,\hbox {Hz})$$, (c) extreme acceleration values ($$>100m/s^2)$$, or d) too many missing values. Recordings that pass the pre-rejection step are resampled to 100 Hz using polyphase resampling. A 5-s segment is trimmed from both ends of the signal as a rough solution to remove the segments that correspond to gestures of picking up or hanging up the phone. The gravitational component of the acceleration is also removed using a high pass FIR filter of order 512 with cutoff frequency at 1 Hz. The signal is then segmented into non-overlapping windows of 5s (hence, w.r.t Fig. 1, the segment length in samples, *W*, equals 500 and the number of acceleration channels, *C*, equals 3) and the segments whose energy across channels is below a threshold of $$0.15 (m/s^2)^2$$ are discarded. The surviving segments are added to the pool. After repeating this process for all recordings of a subject, the resulting pool of segments is sorted in descending order according to the segments energy in the frequency band of PD tremor (3–7 Hz^37^) and the top $$K_1=1500$$ segments are kept, resulting in a PA bag of the form $$X = \{\mathbf {x}_1, \mathbf {x}_2, \dots , \mathbf {x}_{K_1}\}$$, where $$\mathbf {x}_k \in \mathbb {R}^{C\times W}$$ corresponds to an acceleration segment. Bags that contain fewer than $$K_1$$ segments, are zero-padded to reach that length and a masking mechanism is implemented within the model so that only the actual data are used in the computations. On the other hand, subjects that end up with less than five segments in their bag (before the zero-padding step) are discarded altogether. Values for the aforementioned hyperparameters were selected based on previous work^23^.

### Typing dynamics bag creation

Similar to the acceleration modality, the typing data contribution of the subject is summarised as a set of keystroke timing information, i.e. the typing dynamics bag. In this case, each contributed recording corresponds to one instance in the TD bag. A single data recording includes the press and release timestamps of keys tapped during a typing session, with a session defined as the interval between a foreground appearance of the software keyboard and its subsequent disappearance. Given the press and release timestamps of a session, the first step is to compute the Hold Time (HT) for each key and the Flight Time (FT) to the next key. Through this process, the series of timestamps is transformed to a series of 2-tuples, each representing a keystroke in the given session. Since the number of keystrokes in each session may be different, an additional step is required, in order to make sure that all the instances in the resulting bags are of the same dimensions. To this end, we keep only typing sessions with at least 40 keystrokes and compute the normalised histograms of the hold and flight times, using 10ms bins in the range of [0, 1]s for hold time and [0, 4]s for flight time (hence, w.r.t Fig. 1 the number of HT bins, $B_{HT}$, equals 100 and the number of FT bins, $B_{FT}$, equals 400). The upper limits for these ranges were selected based on previous work^28^. Ultimately, the two histograms are concatenated to form a single vector. The resulting session vectors are then sorted in descending order based on their keystroke count, and the top $K_2=500$ are kept to form the TD bag of the subject, that is of the form $X = \{\mathbf {x}_1, \mathbf {x}_2, \dots , \mathbf {x}_{K_2}\}$, where $\mathbf {x}_k \in \mathbb {I}^{B_{HT} + B_{FT}}$ corresponds to the concatenation of the HT and FT histograms of a session. Similarly to the PA bag creation process, any bags that contain fewer than $K_2$ instances are zero-padded up to that length and a masking mechanism is used to disregard the padding in the computations. Finally, we impose a minimum contribution of five valid typing sessions (with $>=40$ keystrokes) for a subject to be considered in our experiments.

### Problem formulation

We formulate the problem of detecting PD and two of its most relevant motor symptoms as a Multiple-Instance Learning (MIL) problem^38^. MIL is a supervised learning framework that allows us to learn mappings of the form $f: 2^{\mathbb {R}^N} \mapsto \{1, \dots , C\}$, that is, mappings of an entire set $X=\{\mathbf {x}_1, \mathbf {x}_2, \dots , \mathbf {x}_K\}$ with $\mathbf {x}_i \in \mathbb {R}^N$ to the set of class labels, given only access to the ground truth of the entire set *X*. This learning situation naturally accommodates the needs of a system targeting PD, as not all data contributed by a PD patient are expected to contain signs of the disease. This can attributed to a number of factors, such as the unilateral manifestation of symptoms combined with operating the smartphone with the unaffected hand, the use of symptom-alleviating medication or the inherent intermittence of some symptoms, like tremor.

We adopt the attention pooling mechanism that was proposed for the MIL task^39^. This mechanism arises as a specific case of a general theoretic result^40^, stating that any permutation-invariant set function can be described as a sum-decomposition of the form $f(X) = \rho (\sum _{x \in X}\phi (x))$ for some suitable transformations $\rho , \phi$. Stepping on this result, the incorporation of weights in the sum operation was proposed^39^:

1 $$\begin{aligned} f(X) = \rho (\mathbf {z}) = \rho \left( \sum _{k=1}^{K} a_k \mathbf {h}_k\right) = \rho \left( \sum _{k=1}^{K} a_k \phi \left( \mathbf {x}_k\right) \right) \end{aligned}$$

where the weights $\alpha _k$ are obtained via an additive attention mechanism^41^

2 $$\begin{aligned} a_k = \frac{\exp {(\mathbf {w}^T \tanh {(\mathbf {V}\mathbf {h}_k^T}) } )}{\sum _{k=1}^{K}\exp {(\mathbf {w}^T \tanh {(\mathbf {V}\mathbf {h}_k^T}) })} \end{aligned}$$

In practice, the parameters $\phi , \rho , \mathbf {V}, \mathbf {w}^T$ of the function *f* are modelled using neural networks whose weights are learnt through stochastic gradient optimization techniques (specific details about the neural architectures used in this work are given in Section Component architecture).

Here we apply this approach separately to each data source up to the attention pooling stage and fuse the resulting embeddings ($\mathbf {z}$ vectors) into a unified representation. To that end, we define a mapping $f: (2^{\mathbb {R}^{K_1}},2^{\mathbb {R}^{K_2}}) \mapsto [0, 1]^3$, that maps two sets of maximum length $K_1$ and $K_2$ respectively, to a vector of three elements in the [0, 1] interval. Using the attention MIL framework, the mapping *f* takes the following form:

3 $$\begin{aligned} \begin{bmatrix} p\left( y_{tr} = 1 | X_1, X_2\right) \\ p\left( y_{fmi} = 1 | X_1, X_2\right) \\ p\left( y_{pd} = 1 | X_1, X_2\right) \end{bmatrix} = f\left( X_1, X_2\right) = \rho (\mathbf {z}) = \rho \left( \mathbf {z}_1 + \mathbf {z}_2\right) \end{aligned}$$

where

$$\begin{aligned} X_i = \{\mathbf {x}_{i1}, \mathbf {x}_{i2}, \dots , \mathbf {x}_{iK_i}\}, \quad \quad \mathbf {z}_i = \sum _{k=1}^{K_i} \alpha _{ik} \phi _i\left( \mathbf {x}_{ik}\right) , \quad \quad i \in \{1,2\} \end{aligned}$$

In the above notation, $X_1$ corresponds to the Postural accelerations (PA) bag of a subject and $X_2$ to its Typing Dynamics (TD) bag. As we can see, each modality has its own feature extractor ($\phi$ function) and attention parameters ($\alpha$ coefficients), while the function $\rho$ is common (the final classifier module in Fig. 1). The final output of the function *f* is a vector of length three, where each element denotes the probability that a subject is positive for either tremor, FMI or PD, given their accelerometer and typing dynamics data (represented by $X_1$ and $X_2$ respectively).

### Collection of the SData and GData sets

Data collection was carried out via a dedicated Android application, developed in the context of the *i-PROGNOSIS* project^14^. Two separate studies were conducted that resulted in the two datasets used in this work. In the *GData* study, the data collection application was made available through Google Play store in Germany, Greece, Portugal, United Kingdom, Spain, Austria and Australia, after receiving ethical approval by the responsible ethics committee in each country. Each participating subject installed the app on their own smartphone via this channel and, after providing electronic consent and some metadata such as their PD status, age and gender, used it to provide data for a time period ranging from a few days up to several months. Subjects were free to withdraw their consent or drop out at any time. The pool of subjects that donated their data through this procedure, formed the GData set (Table 1). At the same time, a smaller group of users, limited to Greece, Germany and the United Kingdom, was explicitly recruited in order to serve as a clinically-validated set. This group differed from the GData set in that, in addition to using the data collection app, they also underwent a full clinical examination by a movement disorders expert, following a standardised medical evaluation protocol^42^ that included two UPDRS evaluations, typically separated by a period of 8 months. This group of participants formed the SData set (Table 1), a well-defined set of PD patients and healthy controls, diagnosed by a movement disorders specialist according to the MDS clinical diagnostic criteria for PD^43^. Therefore, for subjects in the SData set, detailed symptom scores were available (in addition to their PD diagnosis).

One core requirement was to capture data passively, so as to enable unobtrusive screening for PD. To that end, data were recorded automatically when subjects performed certain actions with their personal smartphones. Specifically, accelerometer data were captured whenever a phone call was received or made by the subject. An upper threshold of 75s was set for this type of recording in order to preserve battery life. Keystroke timing data were recorded by a custom software keyboard, developed for the needs of the study and bundled with the Android application. Whenever the subject used the custom keyboard to type, two timestamps were captured per key tap, one indicating the down time (the moment that the virtual key was pressed) and the other indicating the uptime (the moment that the key was released). The typed content was never captured. Data captured from both modalities, along with some pseudo-anonymised metadata, were temporarily stored on the smartphone database and eventually uploaded to a remote server, when the phone was deemed to be idle.

In general, there is a discrepancy between the pool of users that contributed accelerometer data and the pool of users that contributed keystroke timing data. This can be attributed to a number of reasons. First of all, subjects were at liberty to disable data collection for either one of the two modalities. Moreover, in order to contribute keystroke timing data, subjects had to activate the custom keyboard and use it as the default keyboard across applications. Certain subjects decided not to proceed with this action, probably due to familiarity with their keyboard of choice. Finally, there were subjects that did not contribute an adequate amount of data in either modality and were consequently not included in the analysis process. Specific contribution requirements for each data source are given in “Postural accelerations bag creation” and “Typing dynamics bag creation” sections. The discrepancy between the two cohorts can be quantified, by cross-examining the content of Tables 4 and 5, that present the demographics for the contributors to each data source separately, against the SData part of Table 1, that presents the demographics of the user pool that provided data from both sources.

**Table 4:** Clinically-assessed subjects that contributed typing data. All values (except count) refer to the population mean, with the standard deviation given inside parentheses.

	Healthy controls	PD patients
Count	9	16
Age	49.6 (8.8)	60.5 (9.3)
Years diagnosed	-	6.8 (3.8)
UPDRS 22 (hand rigidity)	0.2 (0.4)	2.2 (1.7)
UPDRS 23 (alternate finger tapping)	0.2 (0.6)	2.0 (1.5)
UPDRS 31 (body bradykinesia)	0.2 (0.4)	1.1 (0.5)
Total UPDRS-III score	1.7 (2.4)	20.3 (14.2)

**Table 5:** Clinically-assessed subjects that contributed acceleration data. All values (except count) refer to the population mean, with the standard deviation given inside parentheses.

	Healthy controls	PD patients
Count	29	48
Age in years	57.3 (11.5)	63.3 (9.1)
Years diagnosed	-	7.5 (4.1)
UPDRS 16 (self-report tremor)	0.1 (0.3)	1.1 (0.8)
UPDRS 20 (hand rest tremor)	0.0 (0.2)	1.1 (1.2)
UPDRS 21 (hand action tremor)	0.0 (0.0)	1.0 (1.3)
Total UPDRS-III score	1.4 (2.7)	18.9 (10.7)

### Training details

Due to the nature of the data collection process (Section Collection of the SData and GData sets), some subjects contributed data for only one of the two modalities. Thus, in order to better utilise all the available data, we perform an initial pre-training step, during which, the two distinct branches of the overall model (acceleration and typing branch in Fig. 1) are trained separately on the entire pool of SData subjects that contributed data for that modality, using only the respective symptom label as target. After this pre-training step, the feature extraction and attention modules of each modality are frozen and their initial classifier modules are discarded The two branches are merged via summation of their embeddings and a new, common final classifier module with three outputs is introduced and trained from scratch using the subjects that contributed both sources of data, i.e., the intersection of the two sets of subjects used for pre-training. Training in this stage is performed using a multi-label approach. The total loss function is defined as the sum of the three binary cross-entropy losses for each model output $p(y_i | X_1, X_2)$ against the corresponding target label $y_i$:

4 $$\begin{aligned} \mathcal {L}_{total} = \mathcal {L}_{tremor} + \mathcal {L}_{fmi} + \mathcal {L}_{pd} \end{aligned}$$

where $\mathcal {L}_{i} = -y_{i}\log \big [p(y_{i}|X_1, X_2)\big ]$ and $i \in \{tremor, fmi, pd\}$. The acceleration branch was pre-trained using all subjects that contributed such data (Table 5). As training target we used a binary tremor annotation provided by signal processing experts after examining all accelerometer signals and taking into account the movement disorders expert’s symptom assessment scores (UPDRS-III Item 20 and 21). This procedure was identical to the one used in previous work^23^ and which was found to produce the most reliable tremor label, owing to the many particularities of monitoring tremor in-the-wild (inherently intermittent as a symptom, can be easily missed during UPDRS examination if the latter coincides with a period of good symptom control under PD medication, subject inclined to use the unaffected hand for making phone calls, etc.). The typing branch was pre-trained using data from the cohort of Table (4) and, in addition, a publicly available typing dataset^28^. As the ground-truth for FMI, we used a fusion of the relevant UPDRS Part III item scores and the subject’s self-report of symptoms. Specifically, a subject was considered positive for FMI if they had positive scores for both UPDRS item 22 (the items concerning hand rigidity) and UPDRS item 23 (alternate finger tapping, closely related to bradykinesia) in any one of the two clinical examinations, or if they self-reported signs of fine-motor impairment during the medical history part of the clinical examination. After pre-training, the fused model was finetuned on the subjects that contributed both accelerometer and typing data (Table 1), using the multi-label loss function of Eq. 4. All training steps were performed using mini-batch stochastic gradient descent with the Adam optimizer^44^ and a batch size of 8, as that was the maximum batch size that could fit in the GPU memory. We used the default learning rate of 0.001 during the pre-training step and a reduced learning rate of 0.0005 during finetuning. Experiments were conducted on an NVIDIA 1070Ti GPU with 8GB of memory. Models were implemented in PyTorch (1.1.0).

### Component architecture

The total model consists of three main parts: the feature extraction module, the attention module and the final classifier module. The feature extraction module is specific to each modality. Its goal is to automatically extract useful features from each instance in the input bag. The resulting feature vectors are then pooled together via the attention module. The role of the attention module is to identify the instances that reflect the relevant symptom within the bag (not all instances in a bag do, due to the inherent fluctuations of motor symptoms or the intake of symptom-alleviating medication), and output an embedding in the form of a single vector that summarises the entire bag appropriately. The embeddings of the different modalities are then summed together to produce the subject embedding. Finally, the subject embedding is fed to the final classifier module which outputs the final predictions for tremor, FMI and PD.

For the various components in the model architecture we use standard neural networks architectures. More specifically, for the accelerometer feature extraction module (transformation $\phi _1$) we use a five-layer 1D Convolutional Neural Network (CNN), while for the keystroke timing feature extraction module we use a three-layer fully-connected network. The specific details of both modules are given in Table 6. The attention modules of each modality are modelled using simple fully-connected layers, based on Eq. 2. Finally, the final classifier module $\rho$ is implemented as a three-layer fully-connected network with three outputs, one for each predicted label. Its detailed description is given in Table 7.

**Table 6:** The network architectures used for the feature extraction modules $\phi _1$, $\phi _2$. *k* denotes kernel size, *f* the number of filters in the convolutional layers and *M* the final embedding dimension. $\mathbb {I}$ denotes the unit interval [0, 1].

Feature extractor	$\phi _1(\mathbf {x})$	$\phi _2(\mathbf {x})$
Input	$\mathbf {x}_{1k} \in \mathbb {R}^{3\times 500}$	$\mathbf {x}_{2k} \in \mathbb {I}^{500}$
	3-DOF acceleration	HT, FT hists
Layer 1	Conv1D $k=8, f=32$	Dense $500 \rightarrow 100$
	LReLU ($\alpha = 0.2$)	LReLU ($\alpha = 0.2$)
	MaxPool $k=2$	Dropout $p=0.1$
Layer 2	Conv1D $k=8, f=32$	Dense $100 \rightarrow 50$
	LReLU ($\alpha = 0.2$)	LReLU ($\alpha =0.2$)
	MaxPool $k=2$	Dropout $p=0.1$
Layer 3	Conv1D $k=16, f=16$	Dense $50 \rightarrow M$
	LReLU ($\alpha = 0.2$)	
	MaxPool $k=2$	
Layer 4	Conv1D $k=16, f=16$	
	LReLU ($\alpha = 0.2$)	
	MaxPool $k=2$	
Layer 5	Flatten	
	Dense $320 \rightarrow M$	
Output	$\mathbf {h}_{1k} \in \mathcal {R}^M$	$\mathbf {h}_{2k} \in \mathcal {R}^M$

**Table 7:** Architecture of the final classifier $\rho$. *M* denotes the output dimension of the feature extraction modules, $\phi _1$, $\phi _2$.

Final classifier	$\rho (\mathbf {z})$
Input	$\mathbf {z}\in \mathcal {R}^M$ (see Eq. 3)
Layer 1	Dense $M \rightarrow 32$
	LReLU ($\alpha = 0.2$)
	Dropout $p=0.2$
Layer 2	Dense $32 \rightarrow 16$
	LReLU ($\alpha = 0.2$)
	Dropout $p=0.2$
Layer 3	Dense $16 \rightarrow 3$
	Sigmoid
Output	$p(\mathbf {y}|X_1, X_2)$

### Statistical analyses

For the reported AUC scores in the GData set, we estimated the $95\%$ CIs using a bootstrap approach. First, a subset of $n^\prime$ ($n^\prime =120$) subjects was randomly sampled from the GData set with replacement, and their model scores were used to compute an AUC score. This process was repeated 1000 times and the $95\%$ CIs were computed as the interval between the 2.5 and 97.5 percentiles of the distribution of AUC scores across the 1000 repetitions. Statistical significance testing between the f1-score of the fused and the single-symptom models, was conducted using the independent two-sample *t*-test, assuming equal sample variance, with $n=10$.

### Ethical statement

All research protocols were approved by Ethik-Kommission an der Technischen Universität Dresden, Dres- den, Germany (EK 44022017), Greece, Bioethics Committee of the Aristotle University of Thessaloniki Medical School, Thessaloniki, Greece (359/3.4.17), Portugal Conselho de Ética, Faculdade de Motricidade Humana, Lisbon, Portugal (CEFMH 17/2017), United Kingdom London, Dulwich Research Ethics Committee (17/LO/0909), Comité de Ética de la Investigación Biomédica de Andalucia, Spain (60854c5dbc58dda37b4730edb590a503edbd3572), Sunshine Coast Hospital and Health Service, Australia (41562 HREC/18/QPCH/266) and Studienzentrum der Prosenex AmbulatoriumbetriebsgesmbH an der Privatklinik Confraternitaet, Wien, Austria (002/2018). Recruitment and study procedures were carried out according to institutional and international guidelines on research involving adult human beings. All subjects provided informed consent prior to participating and preserved the right to withdraw from the study at any time.

## Data Availability

The datasets used in this work, will be made publicly available within 6 months of publication, in order to allow enough time for their proper curation.

## Code availability

The source code of the deep learning algorithm (training and inference) will be made available for research uses at https://github.com/alpapado/deep_pd.
